# Genome interpretation in a federated learning context allows the multi-center exome-based risk prediction of Crohn’s disease patients

**DOI:** 10.1038/s41598-023-46887-2

**Published:** 2023-11-09

**Authors:** Daniele Raimondi, Haleh Chizari, Nora Verplaetse, Britt-Sabina Löscher, Andre Franke, Yves Moreau

**Affiliations:** 1 ESAT-STADIUS, KU Leuven, 3001 Leuven, Belgium; 2 Institute of Clinical Molecular Biology, Christian-Albrechts-University of Kiel, Kiel, Germany; 3 University Medical Center Schleswig-Holstein, Kiel, Germany

**Keywords:** Computational models, Genome informatics, Machine learning, Predictive medicine, Medical genetics

## Abstract

High-throughput sequencing allowed the discovery of many disease variants, but nowadays it is becoming clear that the abundance of genomics data mostly just moved the bottleneck in Genetics and Precision Medicine from a data availability issue to a *data interpretation* issue. To solve this empasse it would be beneficial to apply the latest Deep Learning (DL) methods to the Genome Interpretation (GI) problem, similarly to what AlphaFold did for Structural Biology. Unfortunately DL requires large datasets to be viable, and aggregating genomics datasets poses several legal, ethical and infrastructural complications. Federated Learning (FL) is a Machine Learning (ML) paradigm designed to tackle these issues. It allows ML methods to be collaboratively trained and tested on collections of physically separate datasets, without requiring the actual centralization of sensitive data. FL could thus be key to enable DL applications to GI on sufficiently large genomics data. We propose FedCrohn, a FL GI Neural Network model for the exome-based Crohn’s Disease risk prediction, providing a proof-of-concept that FL is a viable paradigm to build novel ML GI approaches. We benchmark it in several realistic scenarios, showing that FL can indeed provide performances similar to conventional ML on centralized data, and that collaborating in FL initiatives is likely beneficial for most of the medical centers participating in them.

## Introduction

In the last two decades^1^, high-throughput sequencing technologies have flooded life sciences with large amounts of genomics data such as Whole Exome (WES) and Whole Genome Sequencing (WGS)^2^. This sudden availability of data initially led to rapid advancements, such as the discovery of causative variants for many Mendelian disorders^3^ and the identification of many associated variants for complex diseases^4,5^. In time, the main bottleneck towards understanding our genome shifted from an issue of data availability to one of *data interpretation*. Despite the growing list of known genetic associations^6,7^ and the attempts at disease risk prediction^8–11^, models that aim to truly capture all the complexity of the underlying biological molecular mechanisms, are indeed still missing^12^.

To fully encompass this the complexity of biology and directly model the genotype-to-phenotype relationship, the idea of applying the latest Deep Learning (DL) methods to genomic data is in principle very appealing. This *genotypes-in, phenotypes-out* predictive paradigm falls under the umbrella term of Genome Interpretation (GI)^13^ and it is very recent, with some of the first GI Neural Network (NN) methods for the case/control prediction of human diseases^14,15^ and the multi-phenotypic regression of quantitative traits^16^ that have been just published.

The recent successes of DL in several fields, such as object and image recognition^17–19^, Natural Language Processing^20^, and molecular biology^21–24^ were all characterized by huge data sets used to train extremely deep architectures. To unlock similar breakthroughs in the genomics and clinical genetics context, researchers thus need a way to aggregate sufficiently large genomics and phenomics^25^ data sets to train data-hungry DL architectures. In a scenario in which clinical and genomics data require large memory storage and are highly privacy sensitive, this aggregation poses several multifaceted issues involving biotechnological, infrastructural, computational, statistical, and even legal and ethical aspects. Standardized, homogeneous and high-quality genomics and phenomics^25^ samples for training and testing ML methods need to be collected, stored, and shared in a controlled and ethical way among collaborators and medical centers^26^. This brings several complications, including statistical issues arising from *batch effects* and other systematic differences^14^ (e.g., technologies, reagents, kits) used in the data acquisition phase by different centers that could make the data not independent and identically distributed (iid). Moreover, sharing the data to create a large centralized data set carries infrastructural issues related to hosting and moving possibly huge amounts of highly sensitive data, that indeed require the highest privacy, protection, and data access control standards^26^.

Federated Learning (FL) is a recently proposed ML paradigm that tackles these issues by allowing advanced ML methods such as DL to be trained and tested in a collaborative and parallel way that does not require the actual exchange of sensitive data between partners^26–28^. FL was originally introduced as a distributed ML paradigm^29,30^ to allow the training of a centralized model with privacy-sensitive data coming from a large numbers of clients (i.e., millions of mobile devices). Due to the similar privacy concerns that sensitive data such as medical imaging, health records and sequencing data carry, recently the FL paradigm has been applied to several life-sciences applications, showing promising results. FL-based data analysis and ML methods have been proposed for the analysis of health records and medical imaging data, such as MRI and fMRI^31–34^, the meta analysis of biomedical data^35^, and the analysis of genomic data^36^, such as gene expression^37^ and Genome Wide Association Studies (GWAS)^38^. The democratization of the access to genomic data through FL is currently being advocated^39^, and FL has been used for the predition of clinical outcomes during the COVID-19 outbreak^40^

In this paper we test the hypotheses that 1) FL is not only a viable framework to perform conventional genomic data analysis, but that it can also be used to train DL GI methods for disease risk prediction and that 2) it can allow multiple centers (i.e. hospitals) with relatively small datasets to pool them together in a privacy-preserving way, benefitting from the resulting larger training data set. To test these hypotheses, we implemented for the first time a “genotype-in, phenotype-out” GI Neural Network (NN) method for the *in silico* exome-based risk prediction of human disorders in the FL context. To do so, we extended and adapted CDkoma, our previously developed GI NN model for the discrimination of Crohn’s Disease (CD) cases from controls^14^, to work within the popular flower FL framework^41^, creating FedCrohn.

FedCrohn is a *proof-of-concept* that demonstrates the feasibility of combining NN GI methods with FL to address the *data interpretation* bottleneck that is hindering clinical genetics and Precision Medicine advancements, by allowing GI NN methods to be trained on larger data sets without actually centralizing the data. FL provides indeed a solution to the several infrastructural, statistical and legal issues that make large genomics data sets hard to gather, currently preventing the application of DL methods to GI.

For this proof-of-concept, we focus on the exome-based risk prediction of CD, which is a subtype of Inflammatory Bowel Disease (IBD)^42^. Its multi-factorial nature reduces the accuracy of sequencing-based disease risk prediction approaches^43^, due to the susceptibility to environmental factors^44^, its variable severity and age of onset^45^. Besides our CDkoma^14^ approach, few similar methods have been developed, in the context of the Critical Assessment of Genome Interpretation (CAGI) challenges^43,46^.

In this paper we use 3 CD data sets from CAGI^43^ to benchmark FedCrohn in different realistic FL scenarios, comparing its performance with CDkoma^14^, which achieves the same goal in non-FL setting (i.e., by centralizing all the data and directly accessing them). We show that NN models for GI have similar performances in FL and non-FL settings, indicating that FL can allow researchers to overcome infrastructural and data sensitivity issues without reducing the model performance. We also show that the number of collaborating FL clients (medical centers providing the data), and thus the level of fragmentation of the data, do not influence negatively FL performance. The objective of this study is to provide a proof-of-concept of FL in realistic GI settings in which each client has non-iid data that show significant batch effects (they are produced in different years and with different technologies) and different case/controls ratios. Our results indicate that (1) FL can learn effective models even in these sub-optimal, but realistic settings, and that (2) performances are in line with the ones that could be obtained in non-FL settings.

## Methods

### Data sets

In this study we used 3 case-control Crohn’s Disease (CD) data sets. They have been respectively used in the 2011 (CAGI2), 2013 (CAGI3), and 2016 (CAGI4) editions of the Critical Assessment of Genome Interpretation (CAGI)^46^ to benchmark the ability of bioinformatics methods to predict CD cases from controls on WES data. The CAGI2 dataset contains 56 exomes (42 cases and 14 controls). As described in^14,43,46^, this data set is peculiar since cases and the controls have been sequenced in different settings, resulting in a striking batch effect between them^14,43^. The CAGI3 dataset contains 66 WES samples (51 cases and 15 controls). Twenty-eight pedigrees and two discordant twin pairs^43^ are recognizable with clustering^46^, but this stratification is less severe than in the CAGI2 dataset. The CAGI4 dataset is the largest and highest quality data set among the three. It contains 111 sequenced exomes (64 cases and 47 controls). All cases are unrelated and only two pairs of controls are related^43,46^. All the data sets are provided as VCF files listing the observed variants. CAGI3 and 4 data sets are mapped onto the hg19 Human genome build, while CAGI2 is mapped onto the hg18 version. More details on these datasets can be found in^14,43^.

### Annotating WES data with Annovar to obtain compact ML-ready feature vectors

The goal of this study is to compare the performance of NN GI models for disease risk prediction implemented in FL and non-FL (conventional) settings. To ensure this comparability, we used the same VCF annotation procedure and feature encoding we proposed in our previous non-FL GI method for exome-based CD risk predictor^14^.

**Figure 1:**
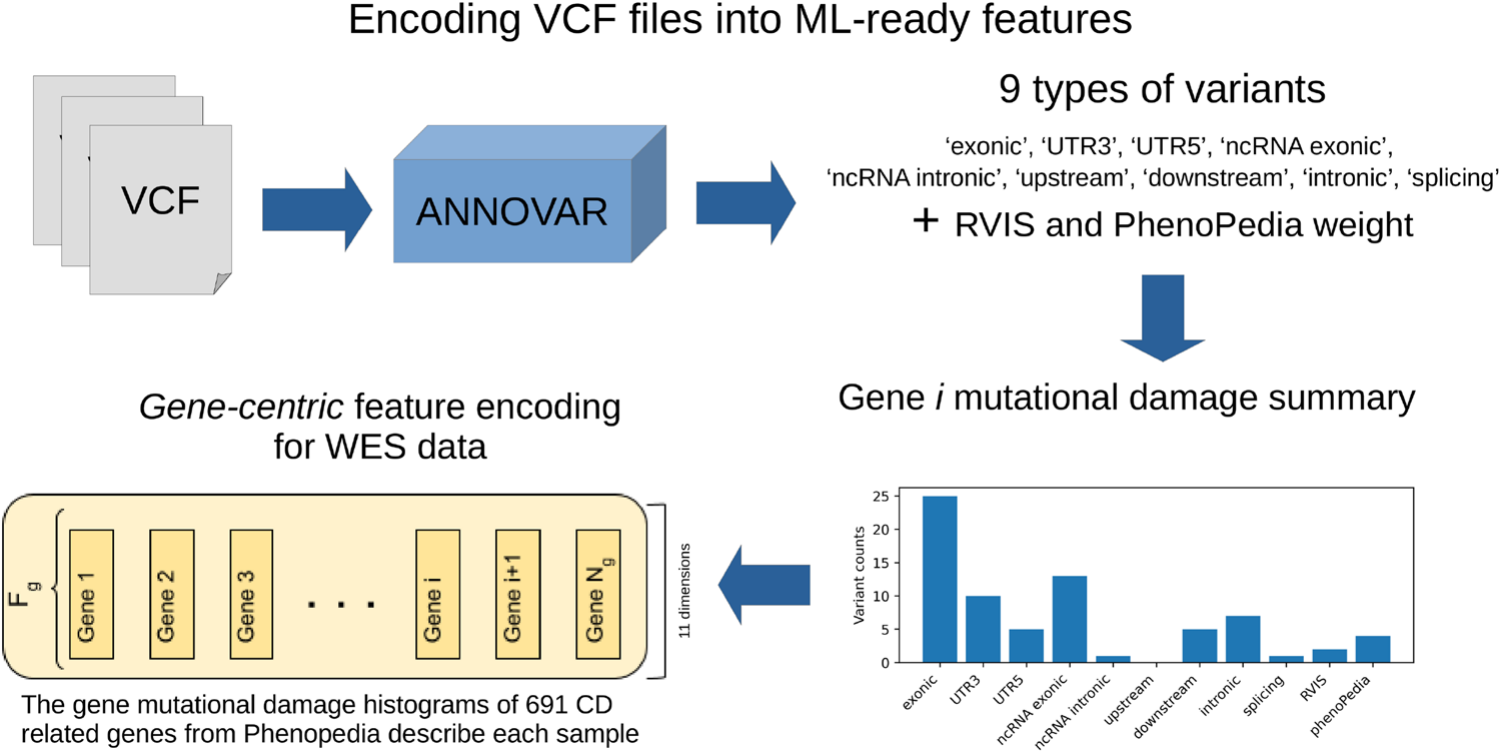
Figure showing the construction of the gene-centric feature encoding used as input for our FedCrohn model. The VCF files from the CAGI datasets are first annotated with annovar. For each gene, its *mutational damage* is summarized by a histogram counting how many times each kind of the 9 classes of variants identified by Annovar are mapped on it. Two gene-level relevance scores (RVIS and the PhenoPedia publication weight) are added to this histogram, obtaining a 11 dimensional vector describing each gene. Each sample is then described by the concatenation of the vectors representing 691 CD-associated genes.

As shown in Fig. 1, we started by annotating all the variants in the CAGI data sets with Annovar^47^, which is a widely used VCF annotation tool. Annovar identified the following 9 types of variants “exonic”, “UTR3”, “UTR5”, “ncRNA exonic”, “ncRNA intronic”, “upstream”, “downstream”, “intronic” and “splicing”.

Given the small sample size of the data sets, we condensed these annotations into the most compact possible ML-ready feature encoding, with the goal of avoiding overfitting by privileging simplicity and model robustness. To do so, we summarized the variants mapped on each gene by counting how many times each type of variant occurs on it, obtaining a histogram describing the *mutational damage* carried by each gene (see Fig. 1). We then concatenated two extra dimensions to each 9-dimensional *feature vector* describing each gene. These two additional dimensions contain the (1) RVIS^48^ gene-burden score and (2) the *publication weight* score extracted from PhenoPedia^49^, obtaining a final vector of 11 dimensions for each gene (see Fig. 1). The intuition behind adding these features is that they should provide some gene-level information to the model, contextualizing respectively the relevance of the gene for human health (RVIS^48^) and its degree of involvement in CD (PhenoPedia^49^).

**Figure 2:**
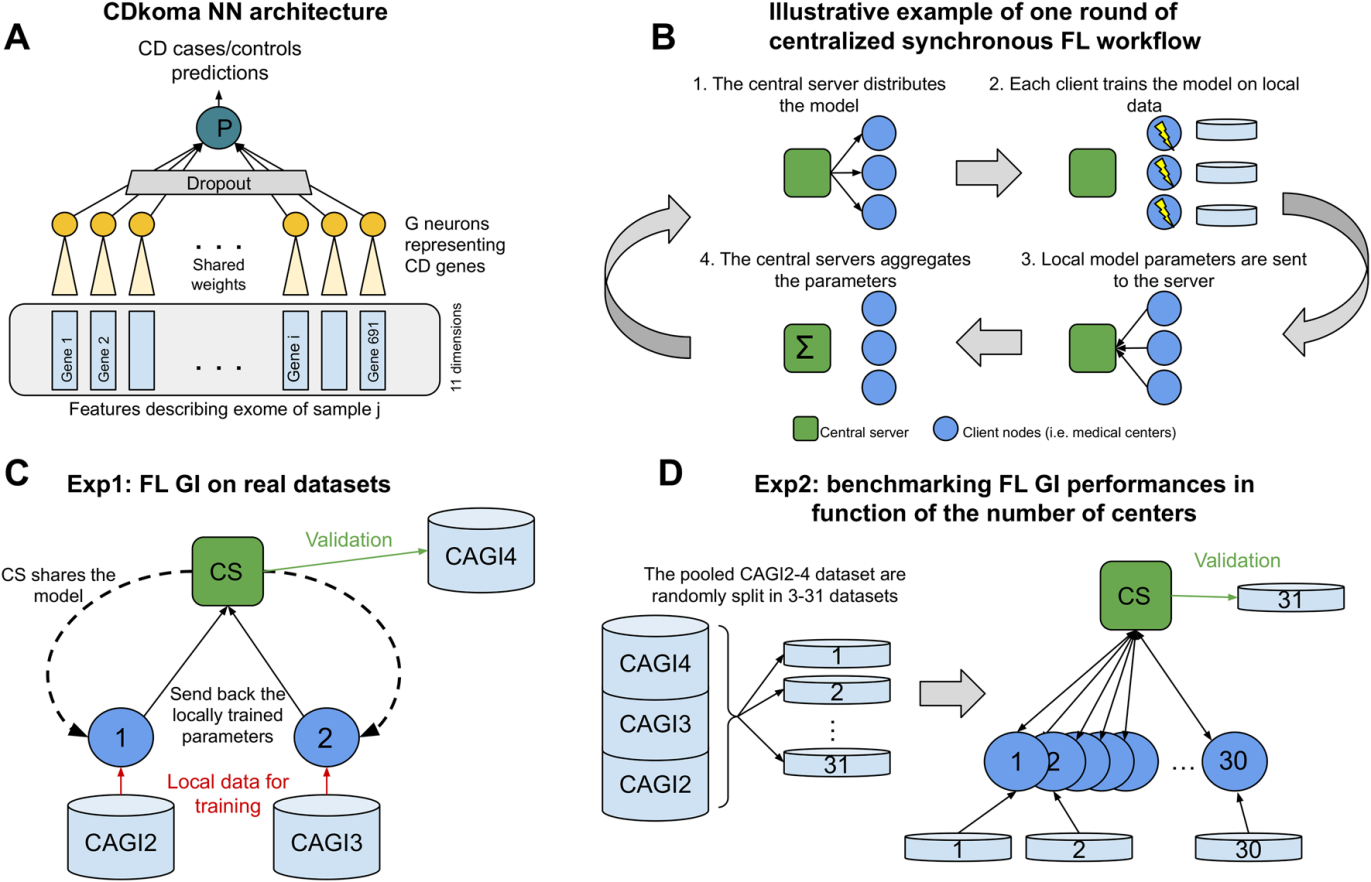
Panel (**A**) shows the CDkoma architecture^14^. Only 691 CD-related genes are considered from each sample’s exome. The mutation burden carried by each gene is read by the shared G neurons, and the final 691 latent values are the input of a logistic regression-like final layer. Panel (**B**) illustrates the workflow of one round of centralized synchronous FL. Panel (**C**) and (**D**) illustrate the two experiments we ran to benchmark the performance of GI FL methods on CD data.

To reduce further the size of this gene-centric feature representation, we considered only 691 CD-related genes, selected from PhenoPedia^49^, instead of the entire exome. Each sample is therefore represented by a (11, 691) tensor, and the final shape of a tensor representing an entire data set containing *N* samples is (*N*, 11, 691), as shown in Figs. 1 and 2A.

### The CDkoma neural network architecture

Genomic data sets tend to have many more features *m* (measured values) than samples *n*, because while sample collection is a relatively slow and complex procedure, WES and WGS data encompass respectively tens of thousands and millions of variants. This heavily underdetermined $m \gg n$ scenario is definitely not ideal for model inference and NNs in particular. To overcome this problem, in our previous GI NN models^14,16^, we reduced the complexity of the models as much as possible by using weight sharing and modular NN structures. Sparsifying the the CDkoma architecture (see Fig. 2A) in this way produces a model with a number of trainable parameters that is proportional to the number of genes in the input features, since the NN module G is shared among all the genes. The G module reads the 11 features describing each gene and summarizes them into a single output value. These values are then concatenated and put through the final layer, that provides a final binary prediction, similarly to a logistic regression (see Fig. 2A). This hierarchical sparse architecture, alongside a Dropout layer with $p=0.1$^50^ and a high *L*2 regularization ($\lambda =1$), limits the ability of the model to overfit the relatively small CAGI data sets.

CDkoma is written in PyTorch^51^. Similarly to^14^, we trained it with the RMSprop optimizer, a learning rate of 0.001, 100 epochs, a batch size of 3 and a binary cross-entropy loss. The small batch size is proportional to the small sample size of the datasets, allowing the network to perform a mini-batch optimization (several weights updates per epoch).

All the hidden neurons used the LeakyReLU activation^52^. The only difference between LeakyReLU and ReLU is that instead of returning 0 for negative activations values ($ReLU(x)=max(0,x)$), the LeakyReLU returns a small negative value instead ($LeakyReLU(x)=max(0.01x,x)$). The intuition behind it is that the small slope for negative activation values can help avoiding the risk of ending up with neurons that are permanently inactive during the training (*dying ReLU* problem).

### The centralized synchronous FL workflow for GI

FL is a distributed inference paradigm in which multiple data-controlling clients (i.e., medical centers) collaborate in training a global consensus model without sharing the possibly sensitive data between each other^26,29^. More formally, given *K* clients controlling privacy-sensitive $(X_k \in \mathcal {X})$ data sets and the parameters $\theta$ of a shared ML model, a global loss function $\mathcal {L}$ is minimized as follows:

1 $$\begin{aligned} \min _\theta \mathcal {L}(\mathcal {X}, \theta ) \text { where } \mathcal {L}(\mathcal {X}, \theta ) = \sum _{k=1}^K w_k L_k(X_k, \theta ) \end{aligned}$$

FL thus optimizes the parameters $\theta$ of a consensus model produced by minimizing linear combination of the training losses of the *K* centers. In this paper, we focus on a centralized synchronous FL involving tens of centers.

As shown in Fig. 2B, in these settings the optimization shown in Eq. (1) is performed via an iterative procedure coordinated by a central node, called Central Server (CS) . First, the CS initializes the model parameters $\theta$ (i.e., random initialization for a NN) and shares them with the *k* clients. Second, each client trains the model $\theta$ on their local data for a certain number of epochs *e*, and then sends the parameter updates resulting from this training to the CS (step 3 in Fig. 2B). In step 4, the CS uses a predefined strategy to aggregate the parameter updates coming from the *k* clients. These 4 steps constitute one round of FL optimization and this is repeated until model convergence. To produce the results shown in this paper, we used 5 FL rounds and 100 epochs for the local training of the GI NN model. The python library we used to implement the federated learning component was flower and the code to reproduce the simulations is available in our git repository at https://bitbucket.org/eddiewrc/FedCrohn/.

### FL parameters aggregation strategies

One of the most crucial challenges of FL algorithms is to combine the local models trained by the *k* clients to form a robust global model through a parameters aggregation strategy.

In more conventional decentralized ML settings, such as distributed learning, the assumption is that the locally distributed datasets belong to the same distribution (i.i.d) and have similar size and labels balancement. The main issue in FL is that none of these assumptions necessarily hold, and the client-controlled datasets are likely to show various kinds of client-specific biases^53^. The aggregation strategies used by the CS need to be robust to these issues and other technical aspects, such as minimizing the number of required communication rounds between clients and the CS^29^.

Different aggregation strategies have been proposed so far, and in this study we will benchmark on five of them: FedAvg^29^, FedAvgM^54^, FedAdam, FedYogi and FedAdagrad^30^. FedAvg stands for Federated Averaging^29^, and it is the most direct translation of conventional Stochastic Gradient Descent (SGD) to FL. In FedAvg, in each FL round the CS collects the model updates (parameters) from the clients and takes their weighted average to create a new global model, which is then shared back to the clients at the beginning of the next FL round. FedAvg may suffer from slow convergence in certain data and class unbalancement scenarios^30,54^, and natural extensions such as FedAvgM (Federated Averaging with Momentum) have been proposed. FedAvgM adds a momentum term to the updates received from the clients, improving the convergence speed and reducing the impact of noisy updates^54^. Extending this even further, in^30^ the authors proposed three CS adaptive aggregation strategies (FedAdam, FedAdagrad and FedYogi) which respectively inspired by the popular Adam^55^, AdaGrad^56^ and Yogi^57^ optimization algorithms. These algorithms further extend the FedAvgM method by keeping track of both the first and second moments of the gradients (i.e. the running average of the gradients and the squared gradients) to adaptively change the learning rate of each individual weight^30^ in function of the (1) sparsity of the gradients and (2) the number of iterations. These approaches are designed to be more robust when where the data distribution across client devices is non-i.i.d^30^. In this paper we benchmark these aggregation strategies using their implementation in the flower library^41^.

### Evaluation of the predictions

We evaluated the performance of FedCrohn using the Sensitivity (SEN), Specificity (SPE), Precision (PRE), Matthews Correlation Coefficient (MCC), Area Under the ROC curve (AUC), and the Area Under the Precision-Recall curve (AUPRC) metrics. In the cross-validations, we computed them for each folds and we averaged them to obtain the final score.

## Results

### FedCrohn: federated learning genome interpretation for the in silico diagnosis of Crohn’s disease

Genome Interpretation (GI) is the umbrella term describing computational methods aiming at modeling the genotype-phenotype relationship^16^. Recently, thanks to the development of flexible Neural Networks (NN) libraries, such as PyTorch, it became possible to develop *ad hoc* NN architectures for different types of problems, adapting the model itself to the structure of the data. This paradigm has shown a lot of potential in several life science fields, with the most prominent example being Alphafold^21^ and structural biology^22–24^ in general.

To apply the same approach to genomics, clinical genetics, and precision medicine, hoping to achieve similar breakthroughs, researchers have to face some specific challenges because of the unique privacy-sensitive nature of clinical data. Currently, infrastructural and legal issues impede the creation of sufficiently large data sets for DL. To overcome this issue, Federated Learning (FL), which is a novel distributed ML paradigm that avoids the necessity of sharing the actual data while training ML models, has been introduced.

We extended our previous CDkoma^14^ NN GI model (see Fig. 2A) for the exome-based discrimination between CD cases and controls in the FL setting, building FedCrohn, which is, to the best of our knowledge, the first attempt at building a “genotype in, phenotype out” GI model in the FL context. We used the 3 CAGI CD datasets (see Methods) and the flower^41^ python library to train and test FedCrohn to simulate different FL scenarios, benchmarking the ability of GI NN methods to be applied in the FL context. We considered two main experimental settings which are described below and illustrated in Fig. 2C,D).

### Exp1: FedCrohn applied on real-case FL for the CD diagnosis

In a real-life situation, medical centers might have relatively small cohorts of sequenced patients. Because of the frequent heavy underdetermination of genomics data, that generally have many more variables than samples, small cohorts are usually not suitable as training data for complex NN models. However, if different centers could pool together their cohorts without centralizing (i.e., sharing the actual data), larger data sets could be available as training sets for data scientists and bioinformaticians.

In Exp1, we simulate exactly this scenario. We use 3 CD case/control cohorts from the 2011 (CAGI2), 2013 (CAGI3), and 2016 (CAGI4) editions of the Critical Assessment of Genome Interpretation (see Methods). They respectively contain 56 exomes (42 cases and 14 controls), 66 exomes (51 cases and 15 controls), and 111 exomes (64 cases and 47 controls). This simulates the situation in which small data sets are scattered among different centers, since the data have been obtained with different data acquisition procedures in different years (see Methods).

In the first FL GI experiment, we thus imagined 2 client nodes (i.e., medical centers, see Fig. 2C) and a Central Server (CS) node. Each of these nodes controls one of the 3 CAGI data sets. The 2 clients use their data to locally train and transmit the parameter updates to the CS, which aggregates them to build a consensus model without seeing the actual data controlled by the centers. The CS then validates the performance on the data set it controls. We repeated this experiment 3 times, to evaluate the performance on each CAGI data set and compare the possible differences.

Each FL round (see Fig. 2B) starts with the CS sharing the model parameters (random initializations in the first round) with the clients. Each client then trains the model received from the CS on its local data, and sends the trained parameters back to the CS. In the last step of a FL round, the CS aggregates the parameter updates received from the clients following a specific strategy, obtaining a consensus model, that will be shared again with the clients at the beginning of the next FL round. Several aggregation strategies have been proposed in literature, and while running these experiments, we benchmarked five of them (FedAvg^29^, FedAvgM^54^, FedAdam, FedYogi and FedAdagrad^30^). See Methods for more details.

#### Evaluation on CAGI2

In Table 1, we show the results obtained when the CS evaluated the FedCrohn model on the CAGI2 data and the 2 clients performed the local training respectively on CAGI3 and CAGI4 data sets. We see that FedAvg produces the highest AUC, but all the aggregation strategies are very similar (within 2 AUC points). The last two rows of Table 1 show the performance of our previously developed non-FL GI model (CDkoma) when it is trained respectively on CAGI3 and 4 and tested on CAGI2. This gives an indication of what performance each center could have obtained by building a GI model on its own data alone, without collaborating towards building a consensus FL model. When predicting CAGI2, both of the clients controlling the CAGI3 and 4 data set would have obtained lower AUCs with respect to the FL consensus model. In particular, the center controlling CAGI3 data would have suffered from 24% lower performance in terms of AUC (Hanley–McNeil test^58^ p-value = 0.048), while the minimal difference in AUC with the center controlling CAGI4 data is not significant ($p=0.41$).

#### Evaluation on CAGI3

Table 2 shows the same experiment, but evaluated on the CAGI3 data. In this case, the adaptive aggregation methods FedYogi and FedAdam outperform slightly FedAvg. Again, when comparing the FL GI model performance with the non-FL CDkoma version trained on the single data sets alone, we see that the center controlling the CAGI4 data would have obtained similar performance (Hanley–McNeil test^58^ $p=0.48$) to the best FedCrohn model (FedYogi), while the center controlling CAGI2 data would have obtained 21% poorer performance ($p=0.022$). Overall the collaboration within the FL framework would have been beneficial for the center controlling the lowest quality data (CAGI2), and indifferent to the one controlling the best quality data (CAGI4)^43^.

#### Evaluation on CAGI4

Table 3 shows the results for the last experiment ran in these settings. In this case, CAGI4 data was used by the CS for evaluating the FL model. Performances are generally lower, because CAGI4 is the highest quality data set among the 3^43^. The best aggregation method is again FedAvg and its variant with momentum FedAvgM. When comparing the AUCs of FedCrohn with respect to the non-FL CDkoma trained on the individual data sets (last two rows of Table 3), we see that the center controlling the CAGI3 data set would have obtained 3.6% higher AUC with a locally trained model^58^ (*p*-value = 0.35), while the center controlling CAGI2 data would have performed similarly to FedCrohn with FedAdagrad, the lowest performing FL method (17% lower AUC with respect to the best FL model). Similarly to the previous experiments, the center with the lowest quality data (CAGI2) would have thus benefited from the FL approach ($p=0.044$). Table 3 is the only setting where an individual center outperforms the best FL approach. As pointed out in^43^, this could be due by the low data quality of CAGI2. The spurious correlations due to batch effects between cases and controls could indeed inject misleading information in the consensus FL model, masking the real genetic patterns associated with CD, thereby decreasing overall performance when this data set is added.

Overall, the three runs of Exp1 thus show that, depending on the quality of the data sets controlled by the centers, the gain obtained by collaborating to build a FL model can vary. Nevertheless, the performance obtained in FL settings are beneficial for all the centers with data set quality below or equal to the average among the collaborating centers, while the performance obtained by the center with the best quality data set are similar to the best FL model, with the highest drop in Table 3 ($-3.6\%$ of AUC).

**Table 1:** Evaluation on CAGI2 data set.

Aggr. method	Sen	Spe	Pre	MCC	AUC	AUPRC
FedAvg	95.32	62.86	88.74	64.04	76.52	88.08
FedAvgM	96.10	61.90	88.57	64.90	76.20	88.23
FedAdam	93.03	61.90	88.23	59.10	75.33	86.90
FedYogi	96.10	61.90	88.57	64.90	74.37	86.00
FedAdagrad	93.80	64.30	88.97	62.60	75.80	86.47
NoFed (CAGI3)	94.60	38.10	82.50	41.80	58.57	75.70
NoFed (CAGI4)	92.23	64.30	88.80	59.13	74.30	86.93

**Table 2:** Evaluation on CAGI3 data set.

Aggr. method	Sen	Spe	Pre	MCC	AUC	AUPRC
FedAvg	81.92	70.66	90.94	50.30	79.10	91.42
FedAvgM	81.40	66.67	89.50	44.00	78.13	90.53
FedAdam	87.17	66.67	90.13	52.87	80.00	88.73
FedYogi	80.77	77.77	92.67	52.53	81.77	88.80
FedAdagrad	80.13	71.13	90.70	47.30	74.83	85.40
NoFed (CAGI2)	92.33	35.57	83.30	33.87	62.67	83.03
NoFed (CAGI4)	95.53	60.00	89.23	61.80	81.30	92.40

**Table 3:** Evaluation on CAGI4 data set.

Aggr. method	Sen	Spe	Pre	MCC	AUC	AUPRC
FedAvg	72.62	67.66	75.70	40.08	71.82	75.26
FedAvgM	72.27	67.40	75.43	39.47	71.57	74.97
FedAdam	47.70	82.97	81.40	32.93	65.17	73.63
FedYogi	39.00	85.10	88.03	30.47	61.70	72.87
FedAdagrad	52.30	70.93	76.40	26.27	59.67	68.07
NoFed (CAGI2)	53.83	66.67	70.17	21.00	59.57	68.43
NoFed (CAGI3)	68.70	77.30	80.93	45.53	74.43	78.80

### Exp2: benchmarking FedCrohn with respect to the number of clients and the data split strategy

**Figure 3:**
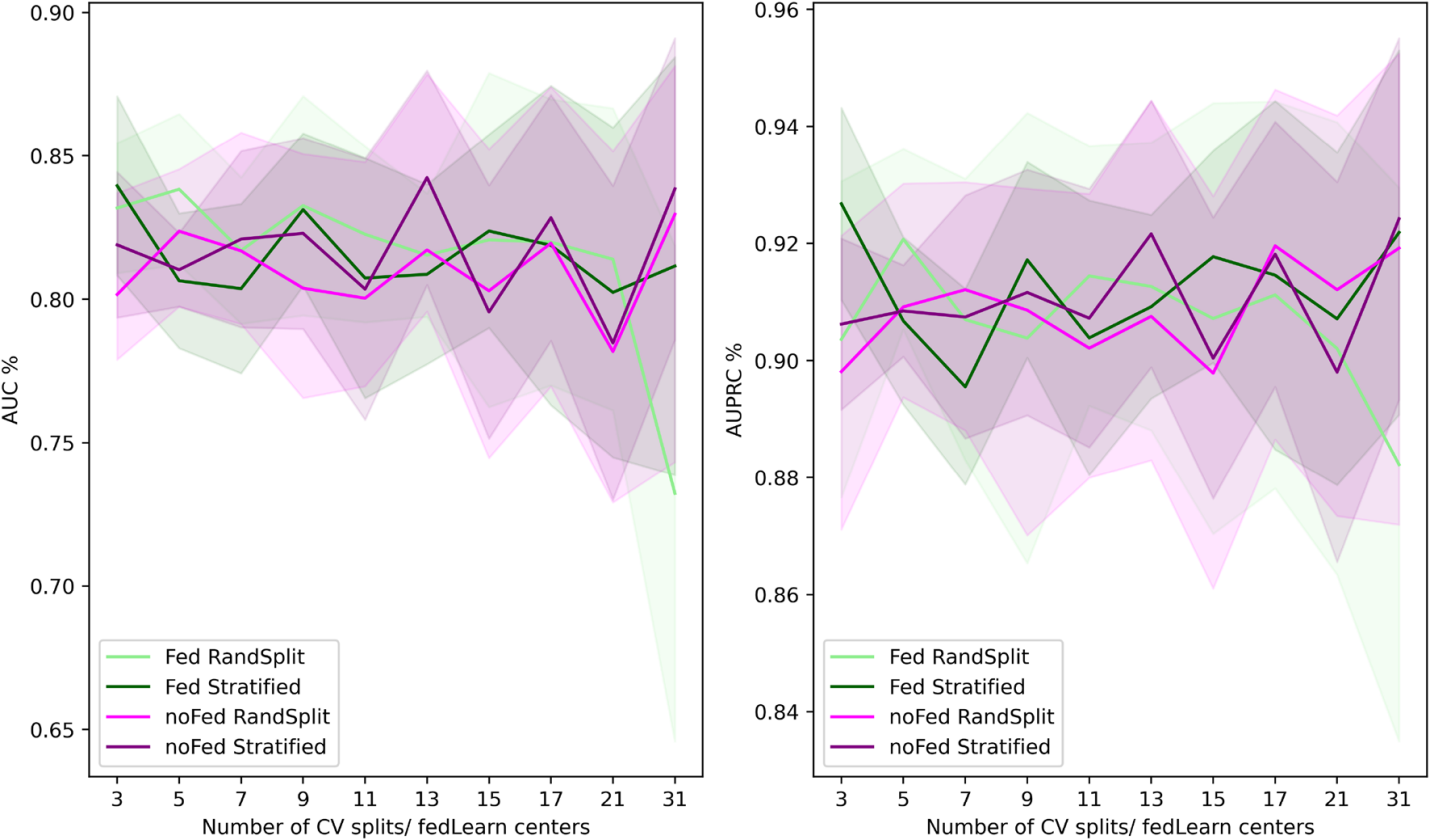
Plot showing FedCrohn performance in function of the number of FL partners involved, compared with non FL models. The shaded areas represent the standard deviations of the measurements..

In Exp2, we investigate FedCrohn performance in function of the number of clients (from 2 to 30) and the way in which the data are divided among them. To do so, we merged the 3 CAGI data sets and we split them in $n = \{3,5,7,9,11,13,15,17,21,31\}$ folds using scikit-learn to obtain (1) random splits and (2) stratified splits that preserve the cases/controls ratio. To run the experiment, we then iteratively held out one fold to be used as validation by the CS and we assigned the remaining to the $n-1$ clients, effectively computing an *n*-fold FL cross-validation (CV).

Figure 3 shows the AUC (left panel) and AUPRC (right panel) performances obtained in function of the number of FL clients/CV splits. To put the FedCrohn scores (light and dark green) in the proper context, we also computed the performance of the non-FL CDkoma model, cross-validated on the same data set splits (light and dark magenta). The colored lines represent the mean AUC and AUPRC scores, while the shaded area represent the standard deviations. From Fig. 3, we can see that the performance of FedCrohn is very similar to the ones obtained by CDkoma, when no FL is involved. In both cases, both AUC and AUPRC is generally high (resp. 85–80 of AUC and 93–89 of AUPRC).

The difference between the random and the stratified splits is that in the latter, the same proportion of positive versus negative cases is guaranteed. When the number of splits gets high, and thus the samples assigned to each fold/center gets lower, stratified splits ensure more stable results, since the random splits might, by sheer chance, assign very few ($\le 1$) samples of a certain class to some folds, thus skewing the predictions from certain folds/clients. The standard deviation of the mean AUC and AUPRC indeed tends to increase with the number of folds, and in particular FedCrohn with 31 randomly split folds produces the lowest performances. The fact that FedCrohn with 31 stratified splits assigned to 31 centers performs similarly to CDkoma suggests that the main driver of this effect is just the positive/negative unbalance in the data sets and not the FL methodology.

Exp2 thus shows that FL GI methods can work on par with respect to non-FL GI approaches regardless of the number of splits, even if many centers providing very small data sets (e.g., 11 samples with 21 folds, 7 with 31 folds) are involved, provided that the negative/positive ratio is more or less preserved.

### Benchmarking FedCrohn against predictors from the past CAGI challenges

In Table 4 we show the comparison of the best FedCrohn performances obtained in Tables 1, 2 and 3 with the models that participated in the previous CAGI 2,3 and 4 challenges. The CAGI official results have been taken from^43^. We also reported the AUC scores of the best performing model in the 2016 edition (CAGI4)^42^. This method, mentioned as “GWAS markers +ML” in Table 4, used ML methods along with CD marker SNPs information from third-party GWAS studies to distinguish between CD cases and controls. We report CDkoma results from the original paper^14^ They are produced by training our model on CAGI4 data to predict CAGI2,3 and by training on CAGI3 data when predicting CAGI4.

**Table 4:** Comparison of FedCrohn AUC scores with the best prediction methods from previous CAGI assessments.

Test set set	Method	AUC
CAGI4	CDkoma$^{\text {c}}$	74
	FedCrohn	72
	GWAS markers + ML $^{\text {a}}$	72
	Ensemble	66
	Manual prediction	63
	Transductive SVM	60
	Key variants weighting	59
CAGI3	Biclustering	87
	Mixed pedigree 1	84
	CDkoma$^{\text {c}}$	83
	FedCrohn	82
	Count of SNVs in CD genes	74
CAGI2	FedCrohn	77
	CDkoma$^{\text {c}}$	74
	Manual prediction	68
	SNV co-occurrence	68
	Biclustering	67
	Count SNVs in CD genes	66

$^{\text {a}}$Result reported from^42^. $^{\text {c}}$

Results reported from^14^. The remaining scores have been taken from^43^.

We briefly summarize the methods listed in Table 4 to provide some context, but they are explained in more details in^43^. The “Key variants weighting” approach consists in ranking the samples in function of the number of known CD-causing SNVs present in the exomes. The “Biclustering” method is a simple K-means clustering of the data with $k = 2$. The “Ensemble” approach is a consensus score combining all the methods described in^43^. “Manual prediction” refers to the manual assessment of each sample, performed by a human expert. The “Count of SNVs in CD genes” produces a score proportional to the variants found on CD-related genes. The “Transductive SVM” approach uses transductive learning^59^ on a set of variants statistically significantly associated with CD^43^.

In Table 4 we show the predictors sorted in function of their AUC scores, which is the metric used by CAGI assessors to benchmark different methods^46^. In all the cases, FedCrohn performs similarly to CDkoma, in line with the results we showed so far, and outperforms most of the approaches benchmarked by CAGI. Nevertheless, we must note that CAGI performances were obtained in true blind test settings. For example, CAGI4 and CAGI3 data was not respectively available to CAGI2,3 and CAGI2 participants. On the other hand, CAGI4 scores are more directly comparable, since CAGI2,3 data were available also to CAGI4 participants.

## Conclusion

The recent astonishing achievements of Deep Learning (DL) methods have been achieved both thanks to the latest developments of Neural Networks (NNs) and to the use of very large training sets. To bring the DL revolution to the Precision Medicine and clinical genetics fields, similarly large genomics and phenomics data collections should be gathered. Thanks to high-throughtput sequencing technologies, data scarcity is not the main factor hindering the creation of such large collections. Instead, due to the high privacy sensitiviy of genomics and phenomics data, several infrastructura, ethical and legal aspects need to be sorted out in order to aggregate smaller datasets into larger studies. Federated Learning (FL) is a distributed Machine Learning paradigm allowing multiple clients controlling different data sets to cooperate towards training a consensus model on the entirety of the data, without actually sharing or moving the data, and thus overcoming many of the above mentioned issues. Here we provide a proof-of-concept (PoC) that FL can be successfully applied to train NNs Genome Interpretation (GI) for the exome-based Crohn’s Disease risk prediction. We test different realistic scenarios, showing that in most cases, the medical centers collaborating towards training a consensus FL GI model, would benefit in terms of quality of the predictions, with respect to the accuracy they could get from a model trained solely on the locally controlled data set. We also show that FL can work even among tens of centers each sharing a very small data set (tens of samples). Our PoC shows that FL could be suitable to kick-start a novel GI paradigm trying to directly model the genotype-phenotype relationship using the latest DL developments.

## Data Availability

The feature vectors the code described in this paper are available at https://bitbucket.org/eddiewrc/FedCrohn/.
